# VEGF overexpressed mesoangioblasts enhance urethral and vaginal recovery following simulated vaginal birth in rats

**DOI:** 10.1038/s41598-023-35809-x

**Published:** 2023-05-27

**Authors:** Marina G. M. C. Mori da Cunha, Bernard K. van der Veer, Giorgia Giacomazzi, Katerina Mackova, Laura Cattani, Kian Peng Koh, Greetje Vande Velde, Rik Gijsbers, Maarten Albersen, Maurilio Sampaolesi, Jan Deprest

**Affiliations:** 1 Group Biomedical Sciences, Centre for Surgical Technologies, KU Leuven, Leuven, Belgium; 2 Group Biomedical Sciences, Woman and Child, Department of Development and Regeneration, KU Leuven, Leuven, Belgium; 3 Laboratory for Stem Cell and Developmental Epigenetics, Department of Development and Regeneration, Stem Cell Institute Leuven, KU Leuven, Leuven, Belgium; 4 Translational Cardiomyology Laboratory, Stem Cell Biology and Embryology Unit, Department Development and Regeneration, Stem Cell Institute Leuven, KU Leuven, Leuven, Belgium; 5 Third Faculty of Medicine, Institute for the Care of the Mother and Child, Charles University, Prague, Czech Republic; 6 Department of Imaging and Pathology, Biomedical MRI/Molecular Small Animal Imaging Center (MoSAIC), KU Leuven, Leuven, Belgium; 7 Laboratory for Molecular Virology and Gene Therapy, Department of Pharmaceutical and Pharmacological Sciences, KU Leuven, Flanders, Belgium; 8 Leuven Viral Vector Core, KU Leuven, Leuven, Belgium; 9 Department of Urology, University Hospitals Leuven, Leuven, Belgium; 10 Pelvic Floor Unit, University Hospitals KU Leuven, Leuven, Belgium; 11 Department of Development and Regeneration, Experimental Gynecology Laboratory –Lok 05.30 ON3, Herestraat 49, Leuven, Belgium

**Keywords:** Adult stem cells, Muscle stem cells, Regeneration

## Abstract

Vaginal birth causes pelvic floor injury which may lead to urinary incontinence. Cell therapy has been proposed to assist in functional recovery. We aim to assess if intra-arterial injection of rat mesoangioblasts (MABs) and stable Vascular Endothelial Growth Factor (VEGF)-expressing MABs, improve recovery of urethral and vaginal function following simulated vaginal delivery (SVD). Female rats (n = 86) were assigned to either injection of saline (control), allogeneic-MABs (MABs^allo^), autologous-MABs (MABs^auto^) or allogeneic-MABs transduced to stably expressed VEGF (MABs^allo-VEGF^). One hour after SVD, 0.5 × 10^6^ MABs or saline were injected into the aorta. Primary outcome was urethral (7d and 14d) and vaginal (14d) function; others were bioluminescent imaging for cell tracking (1, 3 and 7d), morphometry (7, 14 and 60d) and mRNAseq (3 and 7d). All MABs injected rats had external urethral sphincter and vaginal function recovery within 14d, as compared to only half of saline controls. Functional recovery was paralleled by improved muscle regeneration and microvascularization. Recovery rate was not different between MABs^allo^ and MABs^auto^. MABs^allo-VEGF^ accelerated functional recovery and increased GAP-43 expression at 7d. At 3d we detected major transcriptional changes in the urethra of both MABs^allo^ and MABs^allo-VEGF^-injected animals, with upregulation of Rho/GTPase activity, epigenetic factors and dendrite development. MABS^allo^ also upregulated transcripts that encode proteins involved in myogenesis and downregulated pro-inflammatory processes. MABs^allo-VEGF^ also upregulated transcripts that encode proteins involved in neuron development and downregulated genes involved in hypoxia and oxidative stress. At 7d, urethras of MABs^allo-VEGF^-injected rats showed downregulation of oxidative and inflammatory response compared to MABS^allo^. Intra-arterial injection of MABs^allo-VEGF^ enhances neuromuscular regeneration induced by untransduced MABs and accelerates the functional urethral and vaginal recovery after SVD.

## Introduction

Pelvic floor dysfunction (PFD) is a condition with significant physical, mental and social consequences for women worldwide. Childbirth and pregnancy are crucial contributing factors in its pathophysiology. Delivery is a serious traumatic event, causing anatomical and functional changes in the pelvic floor. Passage of the fetal head causes high and sustained pressure and deformation of the pelvic floor^1^ leading to both ischemia and reperfusion and stretch-related injury to nerves, muscles and the supporting structures^2^. Whereas PFD may be present early after delivery, in some women these dysfunctions persist or resurface at a later age. Hypothesizing that symptomatic women may be those who *incompletely recover* morphologically from the trauma caused by vaginal delivery, a secondary prevention strategy by cell therapy administration around the time of the trauma has been suggested in patients with stress urinary incontinence or being at high risk for it^2^, such as previous incontinence, prolonged second stage of labour, high birth weight or any indication of severe trauma evidenced by ultrasound.

In rats, the effects of vaginal birth can be simulated by vaginal distension (VD)^3^ and/or pudendal nerve crush (PNC)^4^. VD mimics the mechanical forces of vaginal delivery and PNC simulates the damage to the pudendal nerve occurring during childbirth. The combination of VD and PNC creates longer lasting functional and anatomical effects^5,6^, whereafter urethral function recovers by 21d postpartum evidenced by the frequency of external urethral sphincter (EUS) bursting^4^. Despite functional recovery, structural changes persist 9 weeks after SVD^5^. Beside urethral injury, SVD also induces structural and functional vaginal damage in rats^4^.

Several studies have been done to document the effects of different stem cell types on pelvic floor function recovery after vaginal birth^7–10^. Administration of stem cells after VD increased urinary LPP as well as the density of vessels, elastin and smooth muscle surrounding the urethra^8,11^. Mesoangioblasts (MABs) are vessel-derived stem cells with a high regenerative potential for muscular disorders^12,13^ and could therefore be good candidates for repairing birth injury. Therefore, we aimed to investigate their effects in a standardized animal model for SVD. Based on our previous findings, we have selected intra-arterial delivery, which resulted in the highest amount of and more homogenous distribution of MABs in relevant pelvic organs^14^.

MABs release several growth factors, such as VEGF, an angiogenic protein with therapeutic potential in ischemic disorders^15^. Moreover, VEGF is also pro-myogenic^16,17^, neuroprotective^18^ and induces nerve regeneration^19^. Therefore, it is reasonable to postulate that increased VEGF secretion by MABs may enhance the regeneration of the pelvic floor structures recovering from vaginal birth injury. The advantages of using genetically modified stem cells as vehicles include the fact that they can ensure a continuous and concentrated local supplementation of diffusible therapeutic molecules, reducing off-target delivery and also the side-effects of systemic administration^20^. MABs show a high adhesion-dependent migratory capacity and can reach perivascular targets especially in damaged areas^21^. Thus, MABs may be effective production sites to ensure stable VEGF delivery, promoting differentiation, survival, and functionality of neurons and muscle cells. Herein we also aimed to evaluate the potential of stable VEGF-expressing MABs in promoting nerve and muscle regeneration of urethra and vagina after SVD injury in rats.

## Materials and methods

### Study design

**Figure 1:**
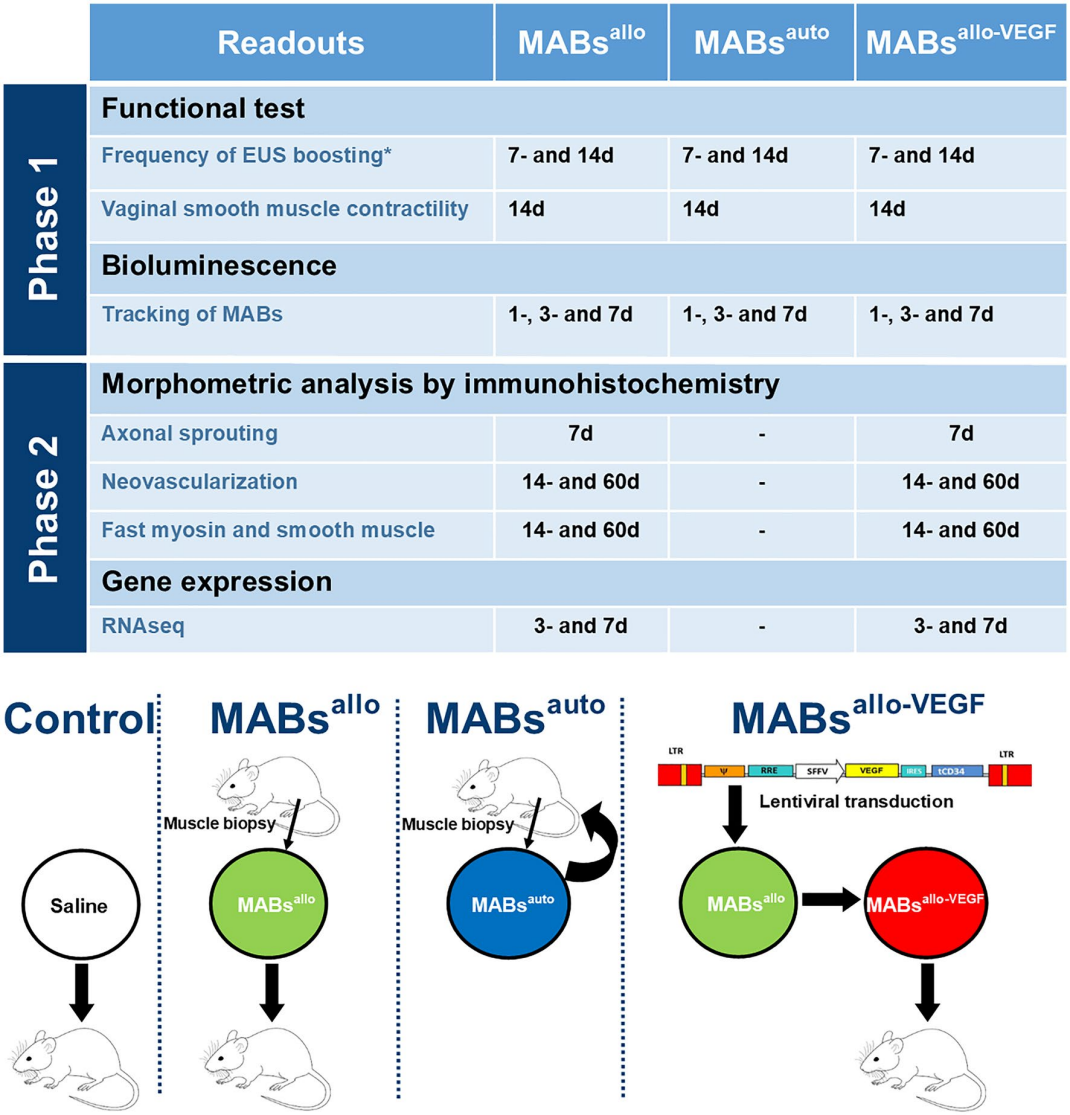
Timeline and design of the experiments: In the MABs^auto^ group, a muscle biopsy is taken at 3-weeks of age, cells are isolated, expanded and re-injected in the same rat 9 weeks later. Control animals are injected with the same volume of vehicle (saline). In the MABs^allo^ group, a muscle biopsy is taken from the gastrocnemius of a 3-weeks old rat and after isolation and expansion, the cell line is injected in a different rat than the donor, who is 12-weeks old. In the MABs^allo-VEGF^ group, the same is done as in the MABs^allo^ group, but cells are transduced with a lentiviral vector encoding VEGF. *MABs*^*auto*^ autologous mesoangioblasts, *MABs*^*allo*^ allogeneic mesoangioblasts, *MABs*^*allo-VEGF*^ VEGF-overexpressed allogeneic mesoangioblasts. Control group: saline injection. *Primary outcome measure.

This study was set up to evaluate the effect of intra-arterial injected MABs in the recovery of the urethral sphincter and the vagina following SVD. The primary outcome was urethral function, evidenced by the frequency of bursting of the EUS during micturition, as measured by micro-ultrasound^22^. This study consists of two phases. First, we investigated how different types of MABs (allogeneic, autologous and VEGF-overexpressed allogeneic cells) boost urogenital functional recovery and investigate their fate in the pelvic region (Fig. 1). Second, we selected relevant groups for further morphological and genetic characterization, to understand the mechanism of the observed functional improvement by VEGF overexpression observed. This study is reported in accordance with ARRIVE guidelines.

### Mesoangioblasts

Skeletal muscles from both hind limbs were harvested, processed and characterized as previously described^14^. To enable tracking MABs after injection in vivo and in real time, and to prove cell viability, MABs were transduced with a lentiviral vector LV_CMV-eGFP-T2A-fLuc. For MABs^allo-VEGF^ treatment, LV_SFFV-VEGF165-T2A-tCD34 (VEGF165 cDNA co-expressed with a truncated form of CD34) was used. Vector constructions were used at 1:100 concentration for 48 h (virus titer 2.34e+08 TU/mL). MABs were subsequently sorted as a GFP^+^ fraction. All vectors are constructed and produced by the Leuven Viral Vector Core. Details are described in S1. Details on the isolation and process of mesangioblasts can be seen in the Supplementary S1.

### Cytokine secretion profiling by cytokine antibody array

Rat Cytokine Antibody Array–Membrane (34 targets/ab133992, abcam, USA) was used to assess the efficacy of transduction and to profile the secretion of MABs^allo-VEGF^ compared to MABs^allo^. Experiment was carried out in accordance with manufacturer’s instructions. Details are described in Supplementary S2.

### Animals: birth injury model and treatments

The animal experiments were approved by the Animal Ethics Committee of the KU Leuven (P271-2015) and performed according to international guidelines. Seventy-four female virgin 12-week-old (250–300 g) Sprague–Dawley rats were used. Rats were anesthetized by intraperitoneal injection of ketamine (70 mg/kg), xylazine (7.5 mg/kg) and buprenorphine (0.05 mg/kg) and SVD was simulated by PNC + VD, as described previously^4,23^. One hour after SVD, rats were randomly assigned to receive an intra-aortic injection of 0.5 × 10^6^ MABs (total volume = 0.8 mL) as described previously^14^. Controls received only saline. Details are described in Supplementary S3.

### Functional analysis

#### External urethral sphincter bursting by micro-ultrasound

Here we proposed the use of micro-ultrasound to assess the function of the EUS because it is a noninvasive and reproducible measurement method. We have previously documented the time course of events and functional recover after SVD using this method^24^. Rats were anesthetized with urethane (Sigma-Aldrich; 1.2 g/kg subcutaneously). During cystometry, the EUS was imaged using the Vevo 2100 micro-ultrasound platform and MS400 (30 MHz) probe (VisualSonics Inc., Toronto, Canada). Bursting activity of the urethral outlet reflects rhythmic opening and closing of the outlet to produce a pulsatile flow of urine^24^. Bursting of the EUS during micturition, which was confirmed by leakage of urine from the meatus, and coinciding with an increase in bladder pressure, was recorded in the motion mode (M-mode). A minimum of three micturition cycles were recorded per animal. Subsequent analysis was performed off-line with the Vevo LAB software (version 3.1.1). The frequency of EUS bursting was calculated and averaged over three cycles for each rat.

#### Vaginal strip contractility

Smooth muscle-strip contractility was determined using a standardized protocol^4^ on the middle segment of the vagina, since that contains more smooth muscle tissue than the other segments. The contractile responses were normalized to tissue wet weight and to the maximum KCl response for the CA protocol. Dose–response curves were fitted and the half maximal effective concentration (EC_50_) was calculated. Details are described in Supplementary S4.

### Bioluminescence in vivo imaging

Cells were tracked in vivo by bioluminescence imaging (BLI) (IVIS Spectrum, Perkin Elmer) and images analyzed with software provided by the manufacturer (Living Image version 4.5). Rats were first anesthetized with 1.5% of isoflurane in 100% of oxygen and then given a single injection containing d-luciferin potassium salt dissolved in PBS (126 mg/kg, SC). Next, consecutive frames were acquired for five minutes until the maximum signal intensity was reached. A region of interest was drawn around the pelvic organs. The total flux (p/s) was measured within this region. BLI data was obtained 1d, 3d and 7d following MABs^eGFP/fLUC^ injection.

### Histology and immunohistochemistry

Whole pelvic floor organs were fixed in formalin, embedded in paraffin, cut into 5 µm sections, and stained with Masson trichrome. Alpha smooth muscle actin (α-SMA) staining was used to assess smooth muscle, Fast Myosin, a marker of type II muscle fibers, CD-34, a marker for endothelial cells, to assess vasculature and GAP-43, an axonal sprouting marker, used to evaluate peripheral nerve regeneration. All morphological analyses were performed using ImageJ software. Details are described in Supplementary S5.

### mRNAseq library preparation and analysis

RNA isolated from urethral tissue (n = 4) was used for mRNAseq libraries using the Lexogen QuantSeq 3ʹ library prep kit and sequenced for a minimum of 1 million SE50 reads per sample at the Genomics Core. Adapter-filtered reads were aligned to Rattus Norvegicus reference genome Rnor6.0 using Hisat2 and gene expression was quantified using HT-seq Count. DESeq2 was used to determine differentially expressed genes. CusterProfiler was used for GO-term enrichment analysis. Details are described in Supplementary S6.

### Statistics

ANOVA statistical tests were performed with Tukey post hoc tests if the data were normally distributed. When not, Kruskal–Wallis statistical tests were performed with Dunn’s post hoc test. Data are presented as mean and SD. Significance was reached when p < 0.05. Data were processed using GraphPad Prism version 5.00 for Windows (Graph Pad Prism, La Jolla, CA, USA).

## Results

### MABs^allo-VEGF^ boost functional urethral and vaginal recovery as compared to MABs^allo^

**Figure 2:**
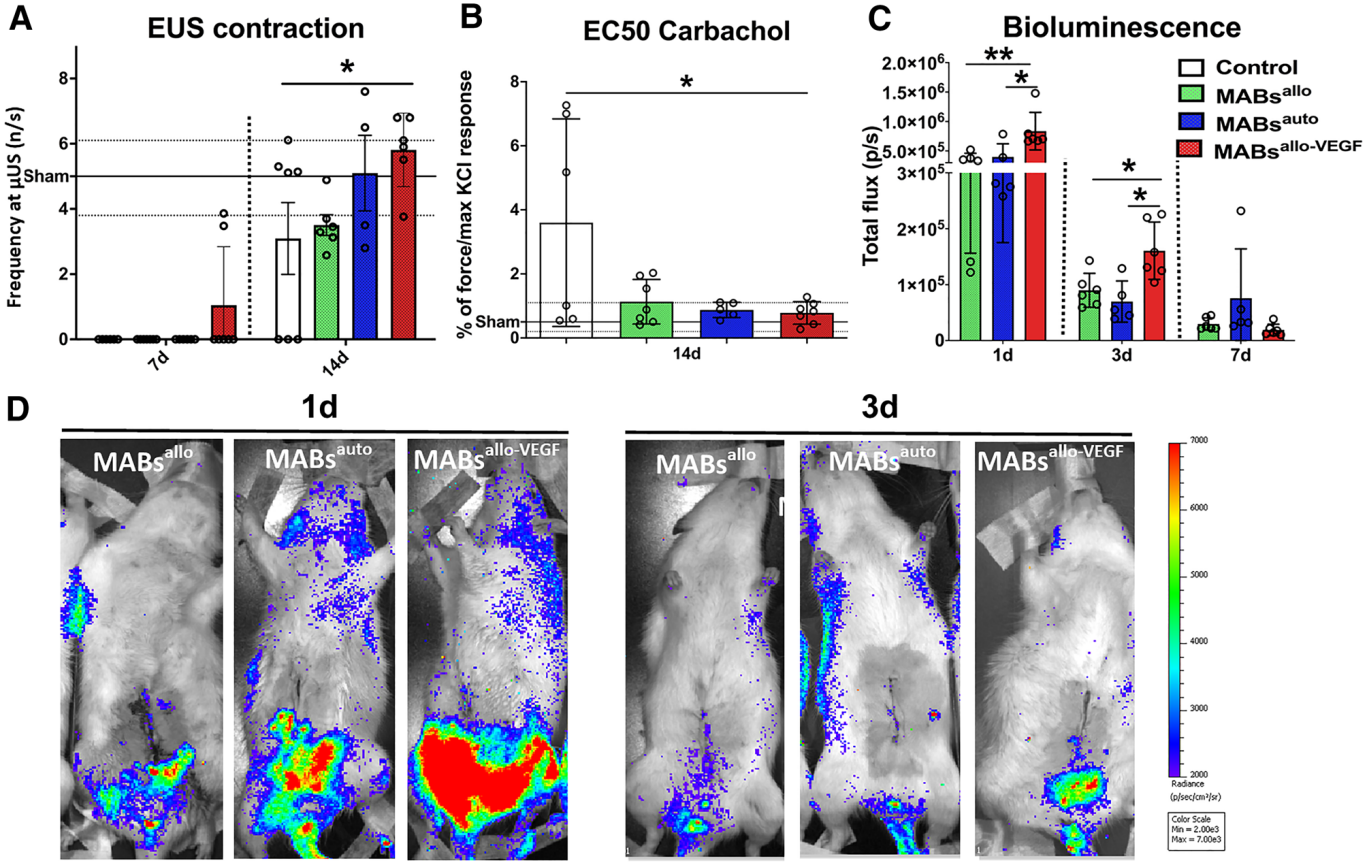
Injection of MABs^allo-VEGF^ accelerates the functional recovery of urethra and vagina, and increases the presence of the cells in the pelvic organs early on: (**A**) Histogram shows the frequency of bursting of the urethral sphincter at 7 and 14d after the injury. There is no measurable function at 7d post-injury, except in MABs^allo-VEGF^ injected rats, of whom 33% show recovery. At 14d, all rats injected with MABs show recovery, as compared to only half of the animals injected with saline. (**B**) Response of the vaginal smooth muscle layer to Carbachol at 14d. Values were normalized to the maximum response to KCl. Vaginal function was recovered in all animals injected with MABs at 14d, compared to only half of the animals in the control group. Only MABs^allo-VEGF^ showed a significantly lower level of EC50 of Carbachol compared to controls. (**C,D**) Longitudinal study of the fate of eGFP/fLUC transduced MABs following intra-arterial administration, as assessed by bioluminescence. (**C**) Histogram shows the signal intensity in the pelvic region at 1d, 3d and 7d. Rats injected with MABs^allo-VEGF^ showed a significantly higher amount of MABs in the pelvic region at 1 and 3d after injection. (**D**) Representative images showing signal in the pelvic region 1d and 3d after injection. *p < 0.05, **p < 0.001; ***p < 0.0001. The horizontal black full and dotted line represents the mean, maximum and the minimum of sham values. *MABs*^*auto*^ autologous mesoangioblasts, *MABs*^*allo*^ allogeneic mesoangioblasts, *MABs*^*allo-VEGF*^ VEGF-overexpressed allogeneic mesoangioblasts. Control group: saline injection. Data are shown as mean and StDev and individual data points.

All rats injected with MABs^allo^ functionally recovered by 14d, whereas only half of the controls recovered. Injection with MABs^auto^ did not further improve that recovery (Fig. 2A). MABs^allo-VEGF^ enhanced the secretion of VEGF (5×) compared to untransduced MABs^allo^ as measured by a rat cytokine array (Supplementary Fig. 1). MABs^allo-VEGF^ accelerated urethral function recovery in 33% of the rats at 7d. Only rats injected with MABs^allo-VEGF^ showed a significant improvement of urethra and vaginal functional recovery at 14d (Fig. 2A,B). Bioluminescence imaging (BLI) was used to determine the longitudinal fate of injected cells (Fig. 2C,D). Up to 7d, there was a progressive decay in BLI signal intensity. Animals injected with MABs^allo-VEGF^ displayed a higher photon flux from the pelvis at 1 and 3d compared with MABs^allo^. Due to absence of significantly improvement using MABs^auto^, autologous cells were omitted from further experiments.

### MABs promote muscle regeneration and neovascularization and MABs^allo-VEGF^ additionally boost axonal sprouting

**Figure 3:**
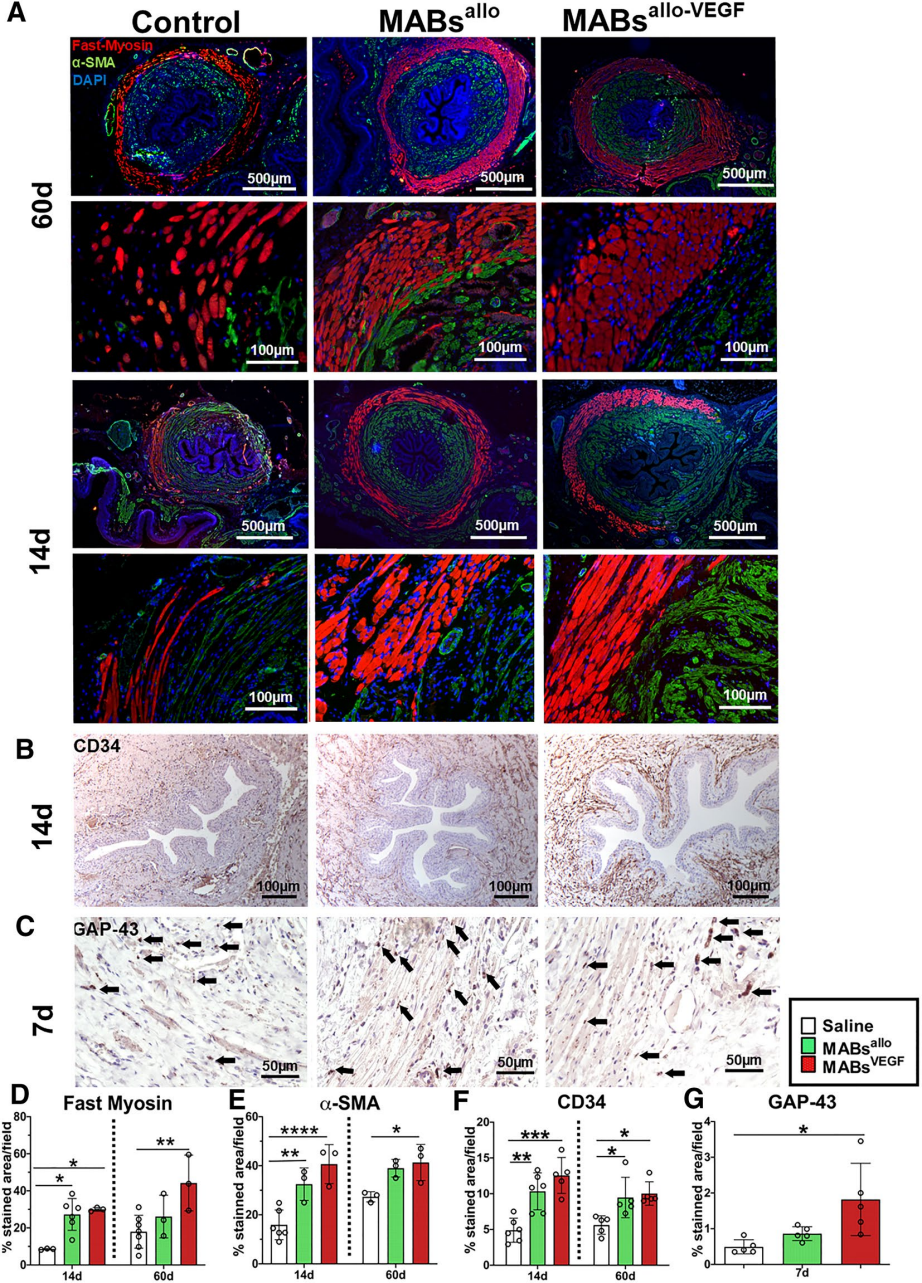
MABs promote myogenesis and neovascularization and MABs^allo-VEGF^ additionally promote axonal sprouting in the urethra following SVD. Representative images of immunostaining for: (**A**) smooth muscle (α-SMA—green) and fast skeletal muscle (Fast Myosin—red) in a mid-urethral section. Nuclei stained in blue (DAPI) at 14d and 60d after SVD; (**B**) endothelial cell marker (CD34) around the mid-urethra at 14d after injection and (**C**) nerve sprouting (GAP-43) around the mid-urethra at 7d after injection. At the bottom of the figure, histograms display counts for (**D**) Fast Myosin, (**E**) α-SMA, (**F**) tCD34 staining at 14d and 60d and (**G**) GAP-43 staining at 7d. At 14d, MABs^allo^ and MABs^allo-VEGF^ injected rats express significantly more Fast Myosin and α-SMA than controls. At 60d, MABs^allo-VEGF^ injected rats express significantly more Fast Myosin and α-SMA than controls. In controls, striated and smooth muscle fibers appear thinner than those injected with MABs at 14d and 60d after injury. MABs^allo^ and MABs^allo-VEGF^ increased neovascularization around the urethra compared to controls at 14d and 60d after injection. At 7d post injury, MABs^allo-VEGF^ rats showed a higher immunostained area for axonal sprouting marker compared to controls. *p < 0.05, **p < 0.001; ***p < 0.0001; ****p < 0.00001. *SVD* simulated vaginal delivery, *MABs*^*allo*^ Allogeneic mesoangioblasts, *MABs*^*allo-VEGF*^ VEGF-overexpressed allogeneic mesoangioblasts. Control group: saline injection. Data are shown as mean and StDev and individual data points.

Morphometric analysis of the EUS was done at 7d (GAP-43), 14d and 60d (Fast Myosin, α-SMA and CD34). Both cell-treated groups showed a significantly higher expression of Fast Myosin and α-SMA, compared to saline injected rats at 14d (Fig. 3A,D,E). Only MABs^allo-VEGF^ increased expression of these markers even further at 60d. Both cell-treated groups also showed increased neovascularization in urethral and vaginal specimens (CD34) at 14d and 60d (Fig. 3B,F, Supplementary Fig. 2A,B). They also showed enhanced axonal sprouting in the vagina at 7d (Supplementary Fig. 2C,D), however, in the urethra this effect was observed only in the MABs^allo-VEGF^ injected rats (Fig. 3C,G).

### MABs^allo^ and MABs^allo-VEGF^ contribute to urethral regeneration early on

**Figure 4:**
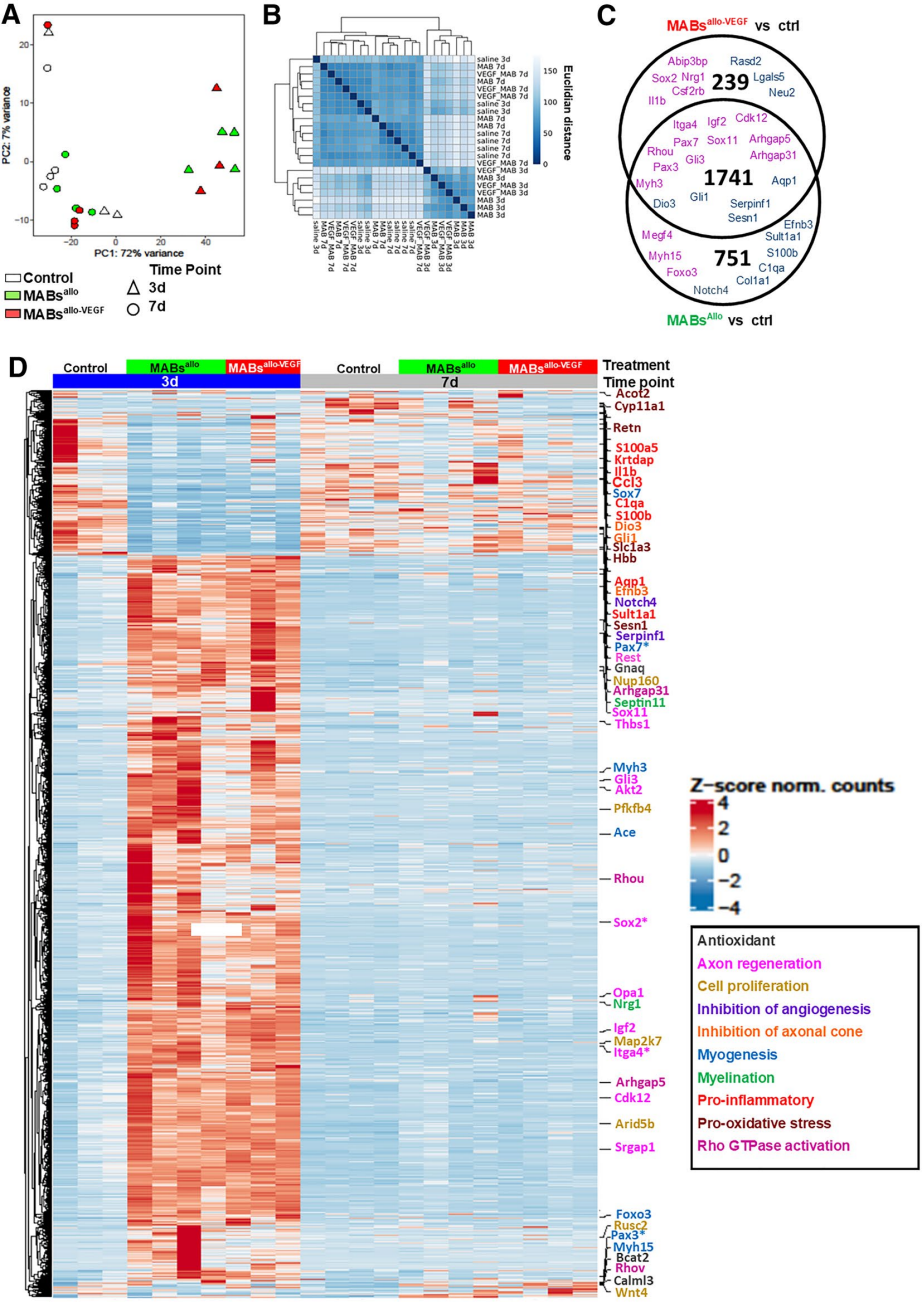
MABs^allo^ and MABs^allo-VEGF^ play a role in slightly distinct pathways 3d after SVD: (**A**) PCA plot of top 500 most variable rlog-transformed genes. MABs treated urethras at 3d post-injury cluster together. (**B**) Hierarchical cluster heat map of Euclidian distance. (**C**) Venn Diagram of the number of DEGs (adj. p.val < 0.05 & |log2(fold-change)| >  1) expressed in each sample at 3d. The intersection shows the overlapping DEGs. There were no DEGs between MABs^allo-VEGF^ vs MABs^allo^. (**D**) Heat map of all DEGs (adj. p.val < 0.05 & |log2(fold-change) |>  1) from rat urethra at 3d and 7d post-injury. *PCA* Principal component analysis, *DEG* differentially expressed genes, *GO* gene ontology, *MABs*^*allo*^ allogeneic mesoangioblasts, *MABs*^*allo-VEGF*^ VEGF-overexpressed allogeneic mesoangioblasts. Control group: saline injection.

To gain a mechanistic insight into how MABs^allo^ and MABs^allo-VEGF^ treatment improves recovery, we analyzed the entire transcriptome from urethra tissue collected 3d and 7d after treatment using mRNAseq. Principal component analysis (PCA) and hierarchical clustering showed that urethra transcriptomes of MABs^allo^ and MABs^allo-VEGF^-3d treated rats clustered, while saline 3d samples clustered with samples collected at 7d (Fig. 4A,B), indicating no significant differences. In line, no differentially expressed genes (DEGs) were found comparing MABs^allo-VEGF^ *vs* MABs^allo^ at 3d. Comparing MABs^allo^ *vs* control and MABs^allo-VEGF^ *vs* control resulted in 2492 (2115 up, 377 down) and 1980 (1787 up, 193 down) DEGs (adj. p.val < 0.05 & log2 (fold change > 1)), respectively, of which 1741 were overlapping (Fig. 4C). A heat map showed that the majority of DEGs were upregulated at 3d in MABs treated rats and were downregulated at 7d and in controls (Fig. 4D).

### MABs promote neuromuscular regeneration and modulate inflammatory response 3d after SVD

**Figure 5:**
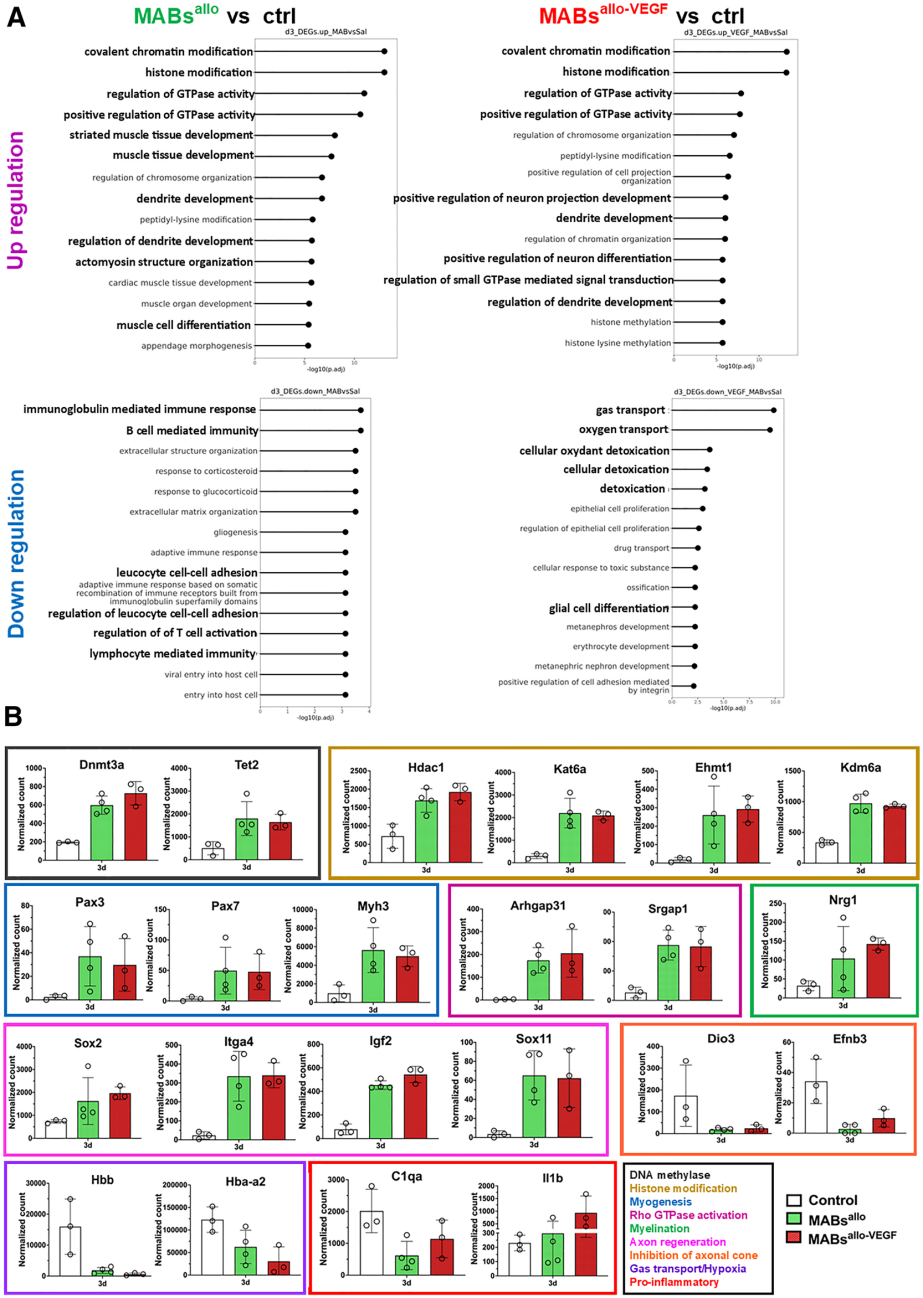
MABs promote neuromuscular regeneration and modulate inflammatory response: (**A**) Top 15 biological process GO-terms associated with upregulated and downregulated DEGs for both the MABs^allo^ vs control and MABs^allo-VEGF^ vs control at day 3. (**B**) Normalized gene count of individual genes involved in DNA methylation (Dnmt3a, Tet2), histone modification (Hdac1, Kat6a, Ehmt1, Kdm6a), myogenesis (Pax3, Pax7, Myh3), Rho GTPase/actin cytoskeleton organization (Arhgap31, Srgap1), myelination (Nrg1), neurogenesis (Sox2, Itga4, Igf2, Sox11), hypoxia (Hbb, Hba-a2) pro-inflammatory (C1qa, Il1b) and inhibition of axonal cone (Dio3, Efnb3). *DEG* differentially expressed genes, *MABs*^*allo*^ allogeneic mesoangioblasts, *MABs*^*allo-VEGF*^ VEGF-overexpressed allogeneic mesoangioblasts. Control group: saline injection. Data are shown as mean and StDev and individual data points.

Top Gene ontology (GO)-terms that were enriched in MABs^allo^ upregulated genes were associated with myogenesis and dendrite development, while the pro-inflammatory process was associated with downregulated genes (Fig. 5A). Top GO-terms enriched in MABs^allo-VEGF^ upregulated genes were associated with neuron and dendrite development, while downregulated genes were enriched in hypoxia and oxidative stress pathways (Fig. 4D). Interestingly, GO-terms enriched in both MABs^allo^ and MABs^allo-VEGF^ were associated with Rho-associated GTPases and epigenetic modifiers. Important up- and downregulated genes in both MABs^allo^ and MABs^allo-VEGF^ are displayed in Fig. 5B.

### MABs^allo-VEGF^ promote actin cytoskeleton reorganization and reduce oxidative and inflammatory response compared to MABs^allo^ 7d after SVD

**Figure 6:**
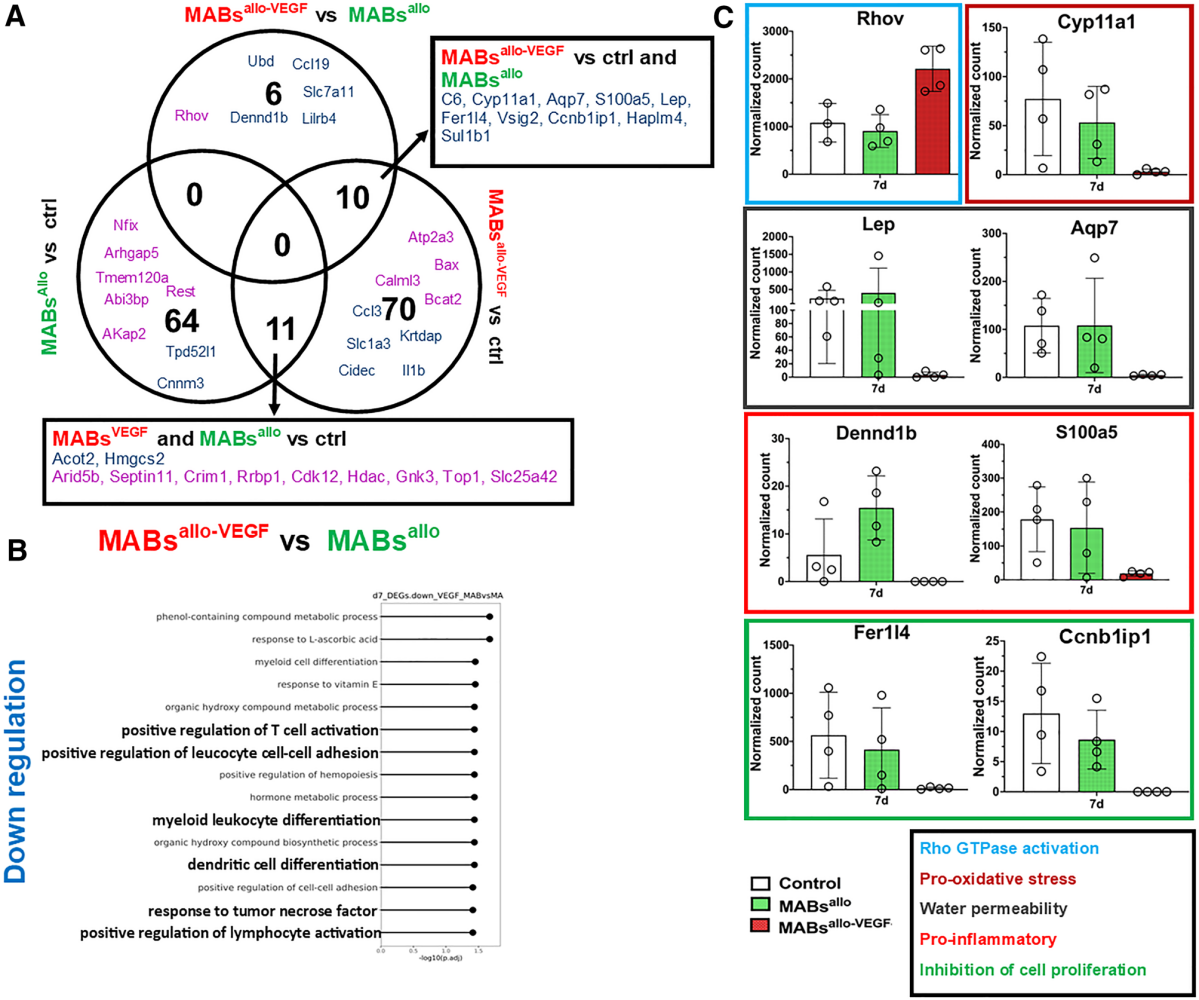
MABs^allo-VEGF^ promote actin cytoskeleton reorganization and inhibit pro-inflammatory response: (**A**) Venn diagram of the number of genes overlapping between each differential comparison performed at 7d post-injury. Purple indicates upregulation and blue indicates downregulation. (**B**) Top 15 biological process GO-terms associated with downregulated DEGs between MABs^allo-VEGF^ vs MABs^allo^. MABs^allo-VEGF^ are involved in the downregulation of pro-inflammatory response. (**C**) Normalized gene count of individual genes involved in Rho GTPase/actin cytoskeleton organization (Rhov), pro-oxidative stress (Cyp11a1), water permeability (Lep and Aqp7), pro-inflammatory response (Dennd1b and S100a5) and cell proliferation inhibitor (Fer1l4 and Ccnb1ip1). *DEG* differentially expressed genes, *MABs*^*allo*^ allogeneic mesoangioblasts, *MABs*^*allo-VEGF*^ VEGF-overexpressed allogeneic mesoangioblasts. Control group: saline injection. Data are shown as mean and StDev and individual data points.

There were 16 DEGs (15 down, 1 up) between the urethra of MABs^allo-VEGF^ *vs* MABs^allo^ at 7d (Fig. 6A), where GO showed the downregulated genes were mainly involved in immune system activation (Fig. 6B). MABs^allo-VEGF^ upregulated *Rhov* (Rho GTPase activation) compared to MABs^allo^ and downregulated *Cyp11a1* (oxidative stress), *Lep, Aqp7* (water permeability), Dennd1b, *S100a5* (inflammatory response) *Fer1l4 and Ccnb1ip1* (cell proliferation inhibitor) (Fig. 6C).

### Distinct gene signature in animals with functionally recovered urethra at 7d

Rats that recovered had 33 DEGs (3 up and 30 down). Top GO terms that were enriched in recovered urethras showed downregulation of IL1-ß and TNF response (Supplementary Fig. 3A). Recovered urethras showed upregulation of *Arhgap18* (actin cytoskeleton organization) and *Apcdd1l* (negative regulator of Wnt pathway) and downregulation of *Angpt4* (angiogenesis inhibitor); *Cidec* (apoptosis); Aqp7 (water permeability); *Ccl3, Ccl4, CD27, Krtdap, Ankef1* (inflammatory response) and *Noxo1, SLC1a3, Retn, Cyp11a1, Pck1, Plin1, Akr1c3 and Adipoq* (oxidative stress).

## Comment

Herein we measured the neuromuscular regenerative effect of intra-arterial administration of MABs^allo^ and MABs^allo-VEGF^ in rats following SVD. Although rats show spontaneous functional urethral recovery by 21d^4,5^, there is a persistent loss of the periurethral and vaginal microvasculature^4^ and dysfunction in the neuromuscular junction of the EUS^9^. Our most important findings were that injection of MABs induced earlier recovery of the EUS. Full functional recovery was paralleled by improved microvascularization and muscle regeneration in the urethra and vagina. Injection of MABs^allo-VEGF^ fastened recovery (33% of animals at 7d), which was paralleled by enhanced axonal growth and improved the quality of recovery seen by the significant improve of urethral and vaginal function at 14d. Interestingly, MABs^allo-VEGF^ also showed a longer-term effect seen by significantly higher muscle content at 60d compared to controls.

This is the first study reporting the *functional* effect of MABs in the rat model for SVD. Similarly to other studies using stem cells^8,25–28^, we observed urethral function recovery and improvement in neovascularization and muscle regeneration at 14d after intra-arterial MABs injection. This improvement coincides with evidence of wound healing activity early on (3d) on mRNAseq such as upregulation of Rho/GTPase activity, epigenetic factors, muscle and dendrite development. MABs have a regenerative potential for muscular disorders due to their capacity to differentiate into skeletal and smooth muscle.

The low survival and poor engraftment of stem cells is one of the obstacles for effective tissue regeneration^29,30^. We hypothesized that *autologous* MABs would persist longer or engraft more, hence improve functional recovery. Herein we did not observe that, which is in line with earlier experiments^31,32^. Low engraftment has been previously explained by the *unfavorable ischemic* *microenvironment* leading to acute apoptosis^33^ as well as loss of cell-adhesion potential inherent to cell preparation^29^.

The improved recovery of the urethra and the vagina, even with the transient presence of the MABs, suggests that the beneficial effect may be mediated primarily through paracrine effects rather than the engraftment. If so, such an effect may also be achieved by administration of drugs, which activate pathways studied herein such as the Rho/GTPase, epigenetic modifiers or others that mimic the observed downstream effect.

One important soluble growth factor secreted by MABs is VEGF, certainly when dealing with ischemic injuries. Therefore, we assessed the additional effect of VEGF, which sped up EUS recovery, and apparently improved nerve regeneration. This may be because of increased cell survival observed at 1d and 3d post injection since VEGF is known for promoting cell survival in a hypoxic environment^34^. VEGF may also be neuroprotective in ischemic injury, e.g. because of its angiogenic effect or its direct trophic effects on neuronal survival^19^. On the molecular level, urethras of MABs^allo-VEGF^ rats showed up regulation of genes that control neuronal development and small GTPases pathways at 3d. Small GTPases are master regulators of actin cytoskeleton rearrangements, which is important for axonal sprouting^35^. On the other hand, *Sox2*, *Il1b* and *Nrg1,* genes involved in dedifferentiation of Schwann cells^36,37^, were among the few genes that were upregulated only in MABs^allo-VEGF^ group at 3d. It is tempting to assume that the activation of these pathways/genes most probably played a role in faster nerve sprouting of the urethra observed at 7d that resulted to a thicker skeletal and smooth muscle fibers observed at 60d. Similarly, other studies using peripheral nerve injury models demonstrated increased muscle volume in animals efficiently re-innervated^38,39^.

Given the clinical feasibility of using allogeneic MABs^allo^ taken from a biobank and immediate availability, our study aimed to investigate the potential merits of allogeneic cell therapy as a more pragmatic approach in patients exhibiting indications at delivery, such as prolonged second stage of labor, high birth weight, or evident trauma confirmed by ultrasound examination. Although autologous cell therapy is ideal and remains a viable strategy, particularly in patients with early indications of being at high risk for stress urinary incontinence (e.g. prior urinary incontinence), it would require all patients to donate cells in advance. Therefore, exploring the use of allogeneic cells from a biobank offers a more practical alternative, as these cells can be readily obtained when needed without relying on individual patient donations.

One limitation of our study is that we did not provide evidence for a paracrine effect. In future experiments this will be important, because our strategy employs gene-modified cells. Though gene modified MSCs are considered to be safe, amenable for repeated treatments and effective, the strategy is still not applied in clinic. Nevertheless, this is the first study reporting functional effects of MABs in a solid rat model for vaginal birth injury, as well as the added effects of delivering VEGF to the site of the injury. Moreover, we used a robust urethral function as outcome measure, shown earlier to be reliable and operator-independent^24^.

We concluded that Intra-arterial injection of MABs^allo-VEGF^ enhances neuromuscular regeneration induced by untransduced MABs^allo^ and accelerates the functional urethral and vaginal recovery after SVD.

## Supplementary Information

Supplementary Information.

## Data Availability

All RNAseq data is available at https://www.ncbi.nlm.nih.gov/geo/ with Accession Number GSE222561. For accessing the dataset, enter token slcdmyoyphcbbaj into the box.
