# Supplementary material for: VEGF overexpressed mesoangioblasts enhance urethral and vaginal recovery following simulated vaginal birth in rats
Source: Sci Rep. 2023 May 27;13:8622. doi: 10.1038/s41598-023-35809-x (PMC10224946; doi:10.1038/s41598-023-35809-x)
Supplement: Supplementary file 1 — Supplementary Information. [file 41598_2023_35809_MOESM1_ESM.docx]

**Supplementary information**

Marina G. M. C. Mori da Cunha^1,2*^, Bernard K. van de Veer^3^, Giorgia Giacomazzi^4^, Katerina Mackova^1,2,5^, Laura Cattani^1,2^, Kian Peng Koh^3^, Greetje Vande Velde^6^, Rik Gijsbers^7,8^, Maarten Albersen^9^, Maurilio Sampaolesi^4^, Jan Deprest^1,2,10^

^1^ Centre for Surgical Technologies, Group Biomedical Sciences, KU Leuven, Leuven, Belgium

^2^ Department of Development and Regeneration, Woman and Child, Group Biomedical Sciences, KU Leuven, Leuven, Belgium

^3^ Laboratory for Stem Cell and Developmental Epigenetics, Department of Development and Regeneration, Stem Cell Institute Leuven, KU Leuven, Leuven, Belgium

^4^Translational Cardiomyology Laboratory, Stem Cell Biology and Embryology Unit, Department Development and Regeneration, Stem Cell Institute Leuven, KU Leuven, Leuven, Belgium

^5^ Institute for the Care of the Mother and Child, Third Faculty of Medicine, Charles University, Prague, Czech Republic

^6^ Department of Imaging and Pathology/ Molecular Small Animal Imaging Center, KU Leuven, 3000 Leuven, Belgium

^7^ Laboratory for Molecular Virology and Gene Therapy, Department of Pharmaceutical and Pharmacological Sciences, KU Leuven, Flanders, Belgium

^8^ Leuven Viral Vector Core, KU Leuven, Belgium

^9^ Department of Urology, University Hospitals Leuven, Leuven, Belgium

^10^ Pelvic Floor Unit, University Hospitals KU Leuven, Leuven, Belgium

**Supplementary material and methods**

**S1. Mesoangioblasts isolation and transduction**

Skeletal muscles from both hind limbs were harvested, processed and characterized as previously described [1]. Briefly, tissue biopsies were minced in ~2 mm size pieces and plated on collagen coated 6 cm dishes. After 10 to 14 days alkaline phosphatase positive cells were sorted. MABs were cultured at 37°C in a 5% CO_2_, 5% O_2_ humidified incubator in DMEM supplemented with 20% FBS, 1% Pen-Strep, 1% L-glutamine, 1% sodium pyruvate, 1% non-essential amino acids, and 0.2% b-mercaptoethanol, (all reagents from GIBCO, City, State, USA). To enable tracking MABs after injection *in vivo* and in real time, and to prove cell viability, MABs were transduced with a lentiviral vector LV_CMV-eGFP-T2A-fLuc or, in the third experiment, LV_SFFV-VEGF165-T2A-tCD34 (VEGF165 cDNA co-expressed with a truncated form of CD34) constructed at 1:100 concentration for 48 hours (virus titer 2.34e+08 TU/mL), and subsequently sorted as GFP^+^ fraction. All vectors are constructed and produced by the Leuven Viral Vector Core as described earlier[2].

**S2. Cytokine array**

Supernatants from MABs^allo^ and MABs^allo-VEGF^ were collected after 48h of culture. Fresh culture medium was used as control. Protein were extracted using RIPA lysis and extraction buffer. Array membranes, each in separate wells of an 8-well plate, were incubated for 30 min of blocking buffer~~.~~ After the membranes were thoroughly washed with wash buffer I and II, of biotin-conjugated antibodies at 1:1000-fold dilution was added to each membrane, and the mixture was incubated on a rotator at 4^o^ C overnight. Primary antibodies were washed and the membranes were incubated with a 1:1000 dilution of HRP-conjugated streptavidin for 2 hours at room temperature. Proteins were detected by detection buffer C and D provided in kit and signals were captured by CCD camera (.10 min of exposure time). Detection of spots using chemiluminescence were acquired using a Biorad Chemidoc XRS+. The intensity of each dot was quantified using ImageJ with “dot blot analysis” pipeline. The signal was normalised to the average of positive control signals in each membrane for comparison of between arrays.

**S3. Surgical procedure**

***Simulated Vaginal Birth***

The animal experiments were evaluated and approved by the Animal Ethics Committee of the KU Leuven (P271-2015) and were performed according to international guidelines. Sixty-three female virgin Sprague-Dawley rats of 12-week-old (250–300 g) were used. Rats underwent either a simulated childbirth injury by pudendal nerve crush and vaginal distension (PNC + VD; n = 42) or sham (n = 15). Anaesthesia was performed in an induction chamber with 5% of Isoflurane (Iso-Vet^®^; Piramal Healthcare UK Ltd, Morpeth; United Kingdom) and maintained with a combination of ketamine (65 mg/kg) (Nimatek^®^; Eurovet Animal Health B.V.; Bladel; Nederland), buprenorphine (0.05 mg/kg) and xylazine (10 mg/kg) (Xyl –M^®^; VMD; Arendonk; Belgium). After the skin was disinfected with polyvidone iodine 7.5% (Braunol^®^; B. Braun Medical, Machelen, Belgium) the animal was draped in a sterile manner and kept on a heating pad until recovery. To induce PNC injury, an incision was made in the dorsolumbar area; the pudendal nerve was identified in the ischiorectal fossa and crushed twice with a needle holder for 30 sec. For simulated VD, a modified 10Fr Foley catheter was inserted into the vagina and the balloon was inflated to 3 mL for 4 h. All animals were kept on a heating pad during surgery and recovery. For post-operative pain-relief, buprenorphine was administered IP for 2 days (0.1 mg/kg, BID).

***MABs administration***

Animals were kept under general anesthesia during administration of cells with 1.5% isoflurane in 100% oxygen at 1.5 L/min. Cells were administered into the aorta. First a ventral midline laparotomy was performed. Next, the internal iliac was dissected (for the clamp placement) and a loose ligature was placed close to the aorta bifurcation to facilitate the insertion of the needle later on. In order to direct the flow of the injected cells towards the pelvic organs, both internal iliacs were occluded with a vascular clamp for 10–20 s. Antegrade catheterization at a 45°angle was performed using a 33 G needle (Acu-Needle, Acuderm, Fort Lauderdale-FL, USA) directly into the aorta and the treatment was administered. After injection, the needle was removed, and the injection site was compressed with a resorbable collagen membrane (Lyostypt®, B. Braun, Aesculap, Tuttlingen, Germany). The abdominal wall was closed with a 3-0 monofilament polypropylene suture in two layers (Prolene, Ethicon, Zaventem, Belgium). Rats also received heparin (400 UI/kg; IM) 1h before injection of cells, and 1 minute before injection the vasodilator isosorbide dinitrate (1 mg/kg; IV).

**S4. Vaginal strip contractility**

Smooth muscle-strip contractility was determined using a standardized protocol [3]. The middle segment of the vagina was chosen, since that contains more smooth muscle tissue than the other segments. Once the response to KCl reached a plateau, the tissue was washed with Krebs solution three times every 10 min until a stable baseline was reached again. Then the strip was exposed to cumulative doses of a non-selective muscarinic receptor agonist carbachol (CA) (Sigma-Aldrich) starting at 10^-8^ to 10^-4^ M to assess receptor function. The contractile responses were normalized to tissue wet weight and to the maximum KCl response for the CA protocol. Dose-response curves were fitted and the half maximal effective concentration (EC_50_) was calculated.

**S5. Immunohistochemistry and morphometric analysis**

Whole pelvic floor organs were fixed in formalin, embedded in paraffin and cut into 5µm sections. Alpha smooth muscle actin (α-SMA) staining was used to assess smooth muscle, Fast Myosin, a marker of type II muscle fibers, CD-34, a marker for endothelial cells, to assess vasculature and GAP-43, an axonal sprouting marker, used to evaluate peripheral nerve regeneration. After deparaffinization, endogenous peroxidase activity was blocked with 0.5% H_2_O2 in PBS for 20 min at room temperature. Sections were then heated at 98°C for 1 hour in citrate buffer (10 mmol/L, pH 6) or TRIS-EDTA to enhance antigen retrieval. Non-specific binding was minimized by incubating sections in 1% BSA and 2% milk in PBS-0.1% tween 80 for 30 min. Sections were then incubated overnight at 4°C with the primary polyclonal antibodies against α-SMA at 1/500 (ab5694, Abcam, Cambridge, UK); Fast myosin at 1/250 (m4276, Sigma), CD34 at 1/1000 (EP373Y, ab81289-Abcam) and Gap-43 at 1/500 (EP890Y, Abcam). Negative controls included buffer alone. For GAP-43 and CD34 specific labeling was detected with EnVision/HRP Detection Kit (DAKO). The color reaction was developed with 3,3'-diaminobenzidine (Sigma-Aldrich, Diegem, Belgium) and sections were counterstained with Mayer hematoxylin. Sections were then dehydrated through graded ethanol, cleared in xylene, and mounted in dePex (BDH, vWR international, Haasrode, Belgium). For immunofluorescence staining, secondary antibodies used included Alexa 488 and Alexa 594 (1/500, Invitrogen). Nuclei were stained with 4’, 6-diamidino-2-phenylindone (DAPI).

Images were acquired using Axioplan 40 microscope (Zeiss, Oberkochen, Germany) using ZEN2 lite software (Carl Zeiss GmbH). All morphological analyses were performed using ImageJ software. Four to six fields of the urethra and the vaginal wall were assessed per staining (×40 magnification). The digital color images were segmented (color deconvolution plugin) and further binarized in order to measure the percentage of the area stained in brown (GAP-43, CD34). For immunofluorescence, area stained in green was used for α-SMA and red for Fast actin. Results are displayed as percentage of the area stained.

**S6. mRNAseq library preparation and analysis**

Urethral tissue (n=4/group) was homogenized in TRIPURE Isolation Reagent (Roche; Vilvoorde; Belgium) followed by purification with the RNEasy system (Qiagen, Valencia, CA, USA). Libraries were prepared with the Lexogen QuantSeq 3' mRNA-Seq library prep kit according to the manufacturer protocol. Samples were indexed to allow for multiplexing. Library quality and size range was assessed using a Bioanalyzer (Agilent Technologies, California, USA)  with the DNA 1000 kit (Agilent Technologies). Libraries were subsequently sequenced on an Illumina HiSeq4000 instrument. Single-end reads of 50 bp length were produced with a minimum of 1M reads per sample.

Quality control of raw reads was performed with FastQC v0.11.7 (available online at: http://www.bioinformatics.babraham.ac.uk/projects/fastqc). Adapters were filtered with ea-utils fastq-mcf v1.05 (Erik Aronesty (2011), ea-utils: “Command-line tools for processing biological sequencing data”; https://github.com/ExpressionAnalysis/ea-utils). Splice-aware alignment was performed with HiSAT2 [4] against the Rattus Norvegicus reference genome Rnor6.0 with default arguments. Reads mapping to multiple loci in the reference genome were discarded. Resulting BAM files were handled with Samtools v1.5 (Li et al, Bioinformatics, 2009). Quantification of reads per gene was performed with HT-seq Count v2.7.14 [5].

Count-based differential expression analysis was done in R (v4.02) using the package DESeq2 [6]. Reported p-values were adjusted for multiple testing with the Benjamini-Hochberg procedure, which controls false discovery rate (FDR), and log fold changes corrected using “ashr” method [7]. The PCA plot (figure 5A), hierarchical euclidian distance matrix, and normalization of gene-counts were made also using DESeq2. GO-term enrichment was performed using the clusterProfiler package [8]. Heatmaps were made with the ComplexHeatmap package [9].

**References**

[1] M.G.M.C. Mori Da Cunha, G. Giacomazzi, G. Callewaert, L. Hympanova, F. Russo, G. Vande Velde, R. Gijsbers, M. Albersen, M. Sampaolesi, J. Deprest, Fate of mesoangioblasts in a vaginal birth injury model: Influence of the route of administration, Sci. Rep. 8 (2018) 1–10. doi:10.1038/s41598-018-28967-w.

[2] R.G. Ibrahimi,Abdelilah; vande Velde, Greetje; Reumers, Veerle;Jaan Toelen, Irina Thiry, Caroline Vandeputte, Sofie Vets, Christophe Deroose, Guy Bormans, Veerle Baekelandt, Zeger Debyser, Highly Efficient Multicistronic Lentiviral Vectors, Hum. Gene Ther. 20 (2009) 845–860.

[3] G. Callewaert, M.G.M.C. Mori da Cunha, K. Dewulf, M. Albersen, J. Deprest, Simulated vaginal delivery causes transients vaginal smooth muscle hypersensitivity and urethral sphincter dysfunction, (2020) 898–906. doi:10.1002/nau.24295.

[4] D. Kim, J.M. Paggi, C. Park, C. Bennett, L. Steven, Graph-based genome alignment and genotyping with HISAT2 and HISAT-genotype, Nat. Biotechnol. 37 (2020) 907–915. doi:10.1038/s41587-019-0201-4.Graph-Based.

[5] S. Anders, P.T. Pyl, W. Huber, Genome analysis HTSeq — a Python framework to work with high-throughput sequencing data, 31 (2015) 166–169. doi:10.1093/bioinformatics/btu638.

[6] M.I. Love, W. Huber, S. Anders, Moderated estimation of fold change and dispersion for RNA-seq data with DESeq2, (2014) 1–21. doi:10.1186/s13059-014-0550-8.

[7] M. Stephens, False discovery rates : a new deal, (2017) 275–294. doi:10.1093/biostatistics/kxw041.

[8] T. Among, G. Clusters, G. Yu, clusterProfiler : an R Package for Comparing Biological, 16 (2012) 284–287. doi:10.1089/omi.2011.0118.

[9] Z. Gu, R. Eils, M. Schlesner, Genome analysis Complex heatmaps reveal patterns and correlations in multidimensional genomic data, 32 (2016) 2847–2849. doi:10.1093/bioinformatics/btw313.

**Supplementary Figures**


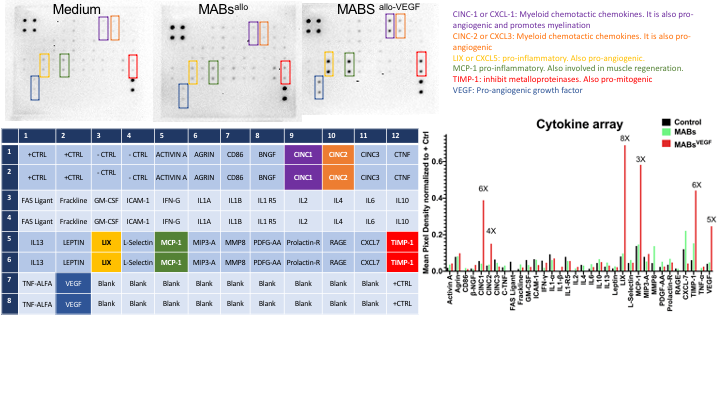


# Supplementary figure 1: Growth factor and cytokine secretion by MABS^allo^ and MABs^allo-VEGF^. MABs^allo-VEGF^ enhanced the secretion of CXCL1 (6x), CXCL3 (4x), CXCL5 (8x), MCP1 (3x), TIMP1 (6x) and VEGF (5x) compared to untransduced MABs^allo^. Targets:  Activin A, Agrin, B7-2/CD86, β (BNGF), C-X-C motif chemokine 1 (CXCL-1 or CINC1), C-X-C motif chemokine 3 (CXCL-3 or CINC2), C-X-C motif chemokine 2 (CXCL-2 or CINC-3), CNTF, Fas Ligand, Fractalkine, GM-CSF, ICAM-1, Interferon γ (IFN-G), Interleukin 1 α and β (IL1a and IL1b), Interleukin 1 receptor 5 (IL1R5), Interleukin 2 (IL2), Interleukin 4 (IL4), Interleukin 6 (IL6), Interleukin 10 (IL10), Interleukin 13 (IL13), Leptin, C-X-C motif chemokine 5 (CXCL5 or LIX), L-Selectin, Monocyte Chemoattractant Protein-1 (CCL2 or MCP-1), macrophage inflammatory protein-3α (MIP3-A), Metalloproteinase 8 (MMP8), Platelet-derived growth factor-AA (PDGF-AA), Prolactin R, Receptor for Advanced Glycation Endproducts (RAGE), CXCL7 (Thymus Chemokine-1), tissue inhibitor of metalloproteinases 1 (TIMP1), Tumor necrosis factor α (TNF-a), Vascular endothelial growth factor (VEGF). Data from 48h cytokine secretion.


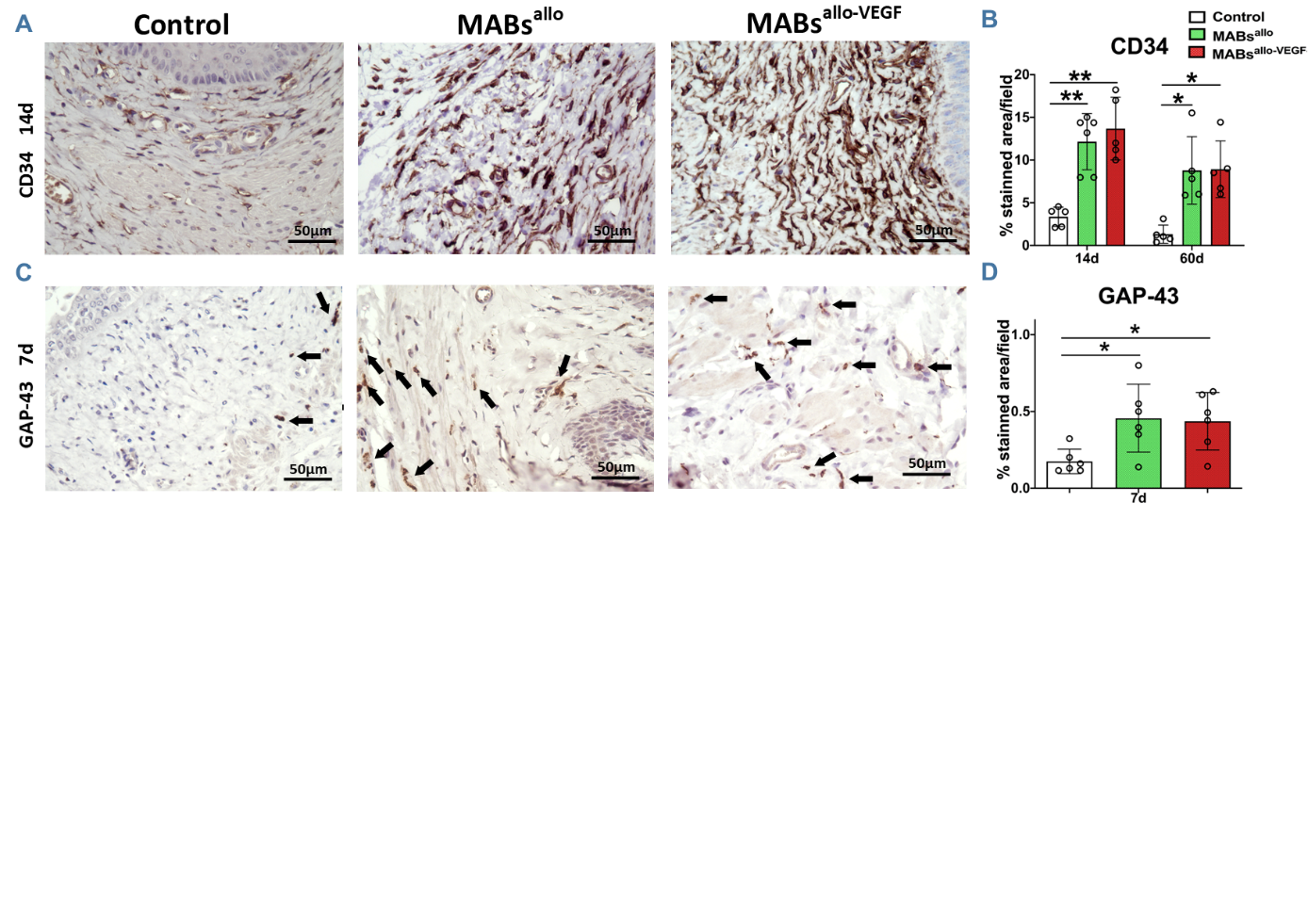


**Supplementary Figure 2.** **MABs improve neovascularization and promote axonal sprouting in the vagina following simulated vaginal birth.** A) Representative images of immunostaining for the endothelial cell marker (CD34) in the mid-vagina. B) Histograms of CD34 morphometric analysis. MABs^allo^ and MABs^allo-VEGF^ increased neovascularization of the vagina compared to controls at 14d and 60d after injection. C) Representative images of immunostaining for nerve sprouting (GAP43) of the mid-vagina at 7d after injection. D) Histograms of GAP-43 morphometric analysis. At 7d, MABs^allo^ and MABs^allo-VEGF^ rats showed a higher immunostained area for axonal sprouting compared to controls. Abbreviations: *p < .05, ** p< .001; MABs^allo^: allogeneic mesoangioblasts; MABs^allo-VEGF^: VEGF-overexpressed allogeneic mesoangioblasts; control group: saline injection. Data are shown as mean and StDev and individual data points.


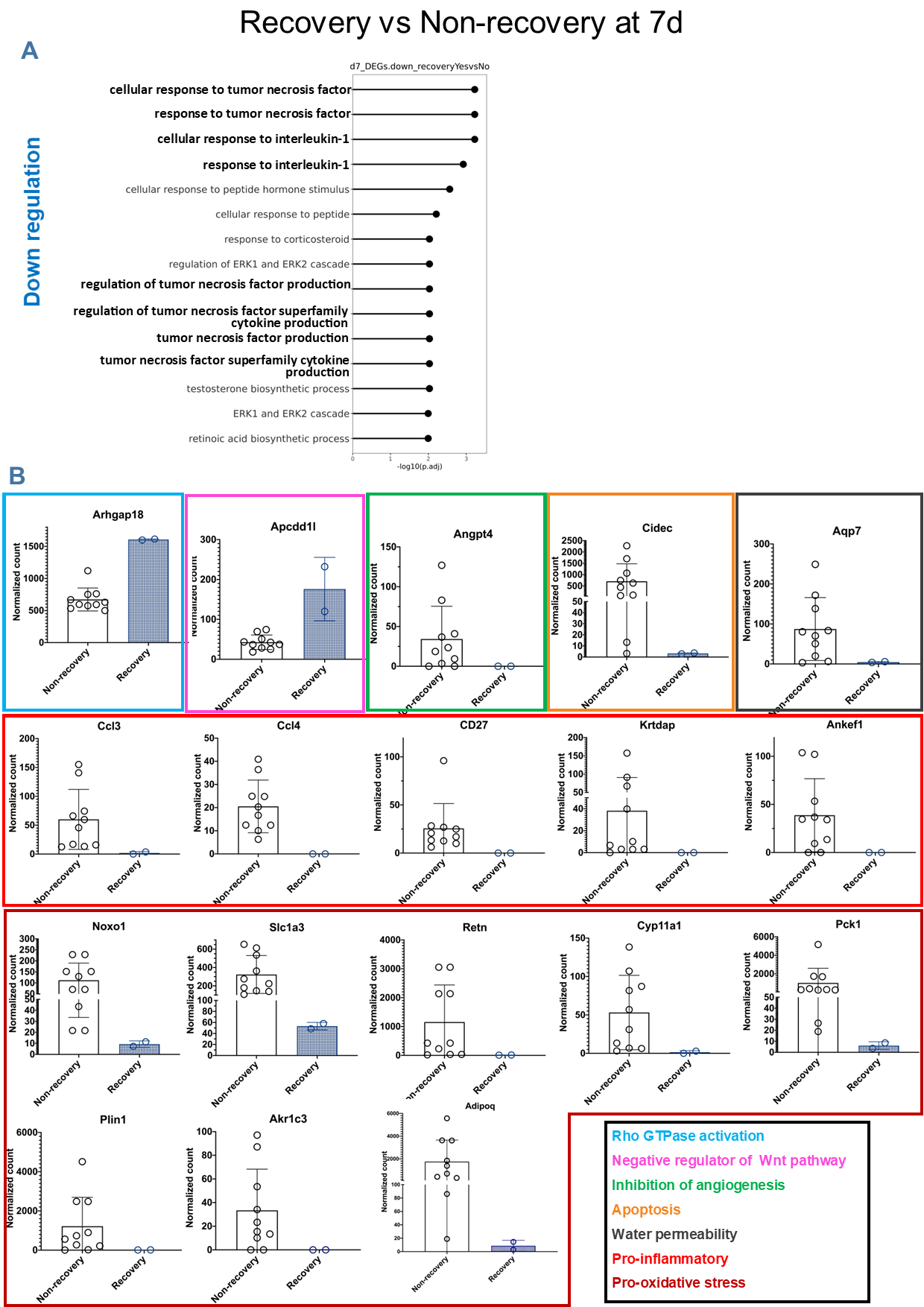


**Supplementary Figure 3. Recovered animals showed modulation of inflammatory and oxidative stress response A)** Top 15 biological process GO-terms associated with downregulated DEGs between non-functionally recovered urethras and functionally recovered urethras. B) Normalized gene count of individual genes involved in actin cytoskeleton organization (*Arhgap18*), negative regulator of Wnt pathway (*Apcdd1l*), inhibition of angiogenesis (*Angpt4*), apoptosis (*Cidec*), water permeability (*Aqp7*), and pro-inflammatory response (*Ccl3, Ccl4, CD27, Krtdap, Ankef1*) and pro-oxidative stress (*Noxo1, SLC1a3, Retn, Cyp11a1, Pck1, Plin1, Akr1c3 and Adipoq*). Data are shown as mean and StDev and individual data points.
